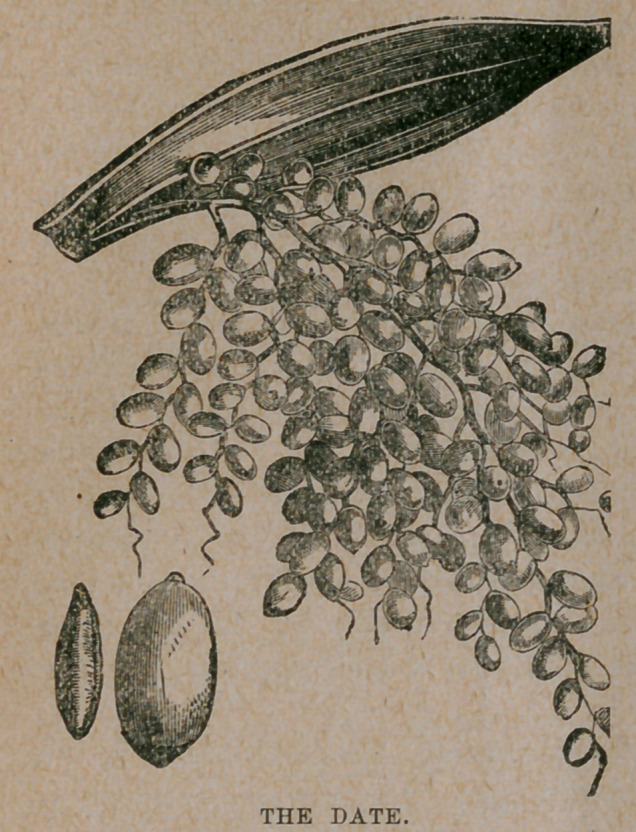# Dates

**Published:** 1889-04

**Authors:** 


### DATES.

The date is the fruit of a tall and graceful palm, *Phoenix dactylifera*, which shoots up a single straight stem to the height of fifty or sixty feet, and then expands into a beautiful crown of leaves. It is abundant in Barbary, Arabia, Persia, and the adjacent countries, particularly on the confines of deserts and in the oases. The fruit somewhat resembles a plum, but is rather longer in proportion; it contains a long oblong kernel, grooved on one side. The pulp is soft, sweet, and slightly astringent: it is rich in sugar, gum, and other vegetable matter, affording the most wholesome nutriment. Dates form a staple article of food to the inhabitants of many countries where they are grown. The Fruit, when gathered quite ripe, is often pressed into large baskets, and thus forms a hard, solid cake, called "adjoue," which is afterward cut up and sold by the pound. Date-stones are soaked in water and given to the cattle.

## Figures and Tables

**Figure f1:**